# Myelin Oligodendrocyte Glycoprotein-Independent Rubella Infection of Keratinocytes and Resistance of First-Trimester Trophoblast Cells to Rubella Virus In Vitro

**DOI:** 10.3390/v10010023

**Published:** 2018-01-04

**Authors:** Quang Duy Trinh, Ngan Thi Kim Pham, Kazuhide Takada, Shihoko Komine-Aizawa, Satoshi Hayakawa

**Affiliations:** Division of Microbiology, Department of Pathology and Microbiology, Nihon University School of Medicine, Tokyo 173-8610, Japan; pham.thikimngan@nihon-u.ac.jp (N.T.K.P.); rsa15836@nifty.com (K.T.); aizawa.shihoko@nihon-u.ac.jp (S.K.-A.)

**Keywords:** rubella, first trimester, trophoblasts, receptor, keratinocytes, infection

## Abstract

Rubella virus (RuV), which belongs to the family *Togaviridae* and genus *Rubivirus*, causes systemic infection in children and young adults and congenital rubella syndrome in developing fetuses if the infection occurs during pregnancy. The mechanisms of fetal infection by RuV are not completely understood. Myelin oligodendrocyte glycoprotein (MOG) is reported to be a cellular receptor for RuV; however, it is mainly expressed in the central nervous system. Therefore, it is thought that other receptors are also responsible for virus entry into susceptible cells. In this study, we found that first-trimester trophoblast cells were resistant to RuV. In addition, we showed that HaCaT cells (an immortalized keratinocyte cell line) that did not express MOG on their surface were infected with RuV. This finding is one of the first demonstrations of MOG-independent RuV infection of susceptible host cells and suggests that it is important to continue searching for alternative RuV receptors. In addition, this study reports the resistance of first-trimester trophoblast cells to RuV and suggests that utilizing an epithelial–mesenchymal transition approach to study the mechanisms of transplacental vertical RuV infection.

## 1. Introduction

Rubella virus (RuV) is an enveloped positive-strand RNA virus in the family *Togaviridae* and genus *Rubivirus* that causes mild symptoms such as rash and fever in children and young adults. However, infections in pregnant women, especially during the first trimester, can lead to dire consequences for the developing fetus, known as congenital rubella syndrome. The mechanisms by which RuV infects the fetus are not completely understood. In addition, many questions related to mechanisms of virus entry into susceptible host cells remain unanswered. Myelin oligodendrocyte glycoprotein (MOG) is reported to be a cellular receptor for RuV [1]; however, it is mainly expressed in the central nervous system and is barely detectable in other organs and tissues. Therefore, it is thought that other receptors are also responsible for virus entry into susceptible host cells [1,2].

While conducting our long-term research focused on the mechanisms of transplacental vertical RuV infection during the first trimester of pregnancy, we became interested in the role of the well-known protein MOG as a receptor for RuV and wondered if first-trimester trophoblast cells are susceptible to RuV. We found that first-trimester trophoblast cells were resistant to RuV. In addition, we showed that HaCaT keratinocytes that did not express MOG on their surface were infected with RuV. This finding is one of the first demonstrations of MOG-independent RuV infection of susceptible host cells and suggests that it is important to continue to search for alternative RuV receptors.

## 2. Results and Discussion

**Figure 1:**
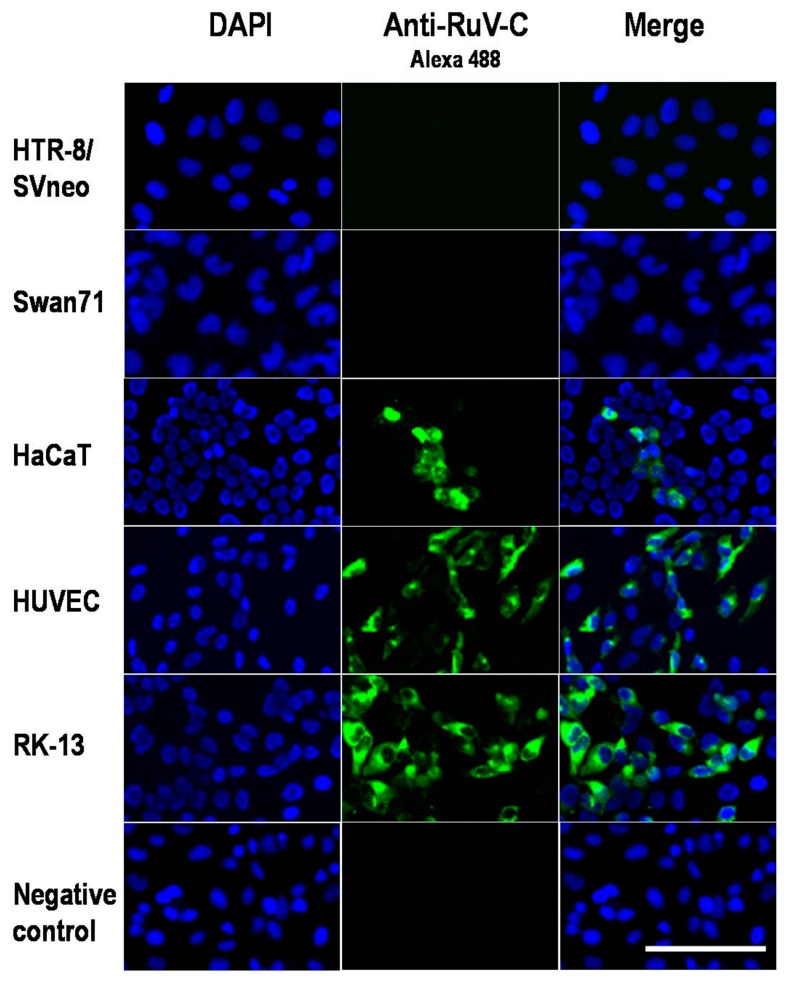
Microscopic images of cells infected or not infected with RuV. The cells were labeled with a mouse monoclonal anti-rubella virus capsid antibody (ab34749, Abcam, Tokyo, Japan) followed by a FITC-conjugated rabbit anti-mouse IgG (H + L) secondary antibody (green); nuclei were stained with DAPI (4′,6-diamidino-2-phenylindole dihydrochloride) (blue). RuV-infected RK-13 cells and HUVECs were used as positive controls, and RuV-infected RK-13 cells stained with mouse serum were used as a negative control. No significant differences were noted on the infectivity of clinical and vaccine RuV strains to the studied cells by immunofluorescence assay. Images are representative of at least three independent experiments. RuV-C, rubella virus capsid. Scale bar: 100 μm.

**Figure 2:**
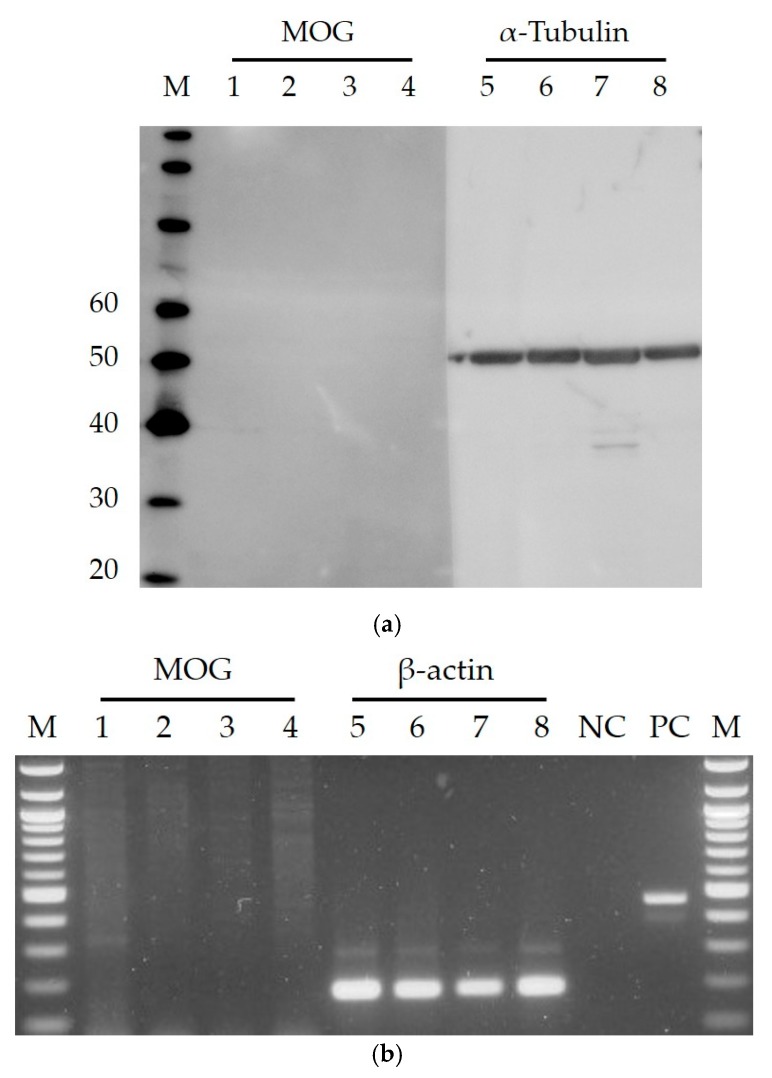
Investigation of MOG expression on first-trimester trophoblast cell lines, HaCaT and HUVECs. Images are representative of at least three independent experiments. (**a**) Western blot analysis. M, molecular weight marker (MagicMark XP Western Protein Standard, Invitrogen, Tokyo, Japan). Lanes 1–4 (for MOG, expected size: 28 kDa) and 5–8 (for α-Tubulin), HTR8/SVneo, Swan71, HaCaT, HUVEC. (**b**) RT-PCR. M, 100 bp marker (TaKaRa, Shiga, Japan); lanes 1–4 (MOG, expected size: 218 bp) and 5–8 (for β-actin, 186 bp), HTR8/SVneo, Swan71, HaCaT, HUVEC; NC, negative control (RNAse-free water); PC, positive control (mRNA and corresponding primers provided in the RT-PCR Kit).

In this study, we found that the first-trimester trophoblast cell lines HTR-8/SVneo and Swan71 were resistant to both vaccine (Takahashi strain) and clinical (3-B1-RK13 strain) RuV strains, while keratinocytes (HaCaT) showed susceptibility to the virus (Figure 1). Nevertheless, MOG was not detected in any of the studied cells by indirect immunofluorescence assays and Western blotting, including human umbilical vein endothelial cells (HUVECs), which were previously reported to be permissive to RuV [3]. In addition, MOG mRNA was not detected by RT-PCR (Figure 2).

RuV was isolated from the skin of rubella patients [4,5]. It has been shown to cause persistent infections in keratinocytes, forming granuloma lesions in patients with primary immunodeficiency [6]. In this study, RuV could infect HaCaT cells, as well as HUVECs, that did not express MOG on their surface. Therefore, receptors other than MOG must be required for virus entry and replication in these cells. Otsuki and colleagues have recently published their findings showing that RuV has two distinct binding mechanisms: a Ca^2+^-dependent mechanism observed in lymphoid cells involving a direct interaction between RuV E1 protein and sphingomyelin/cholesterol-enriched membranes, and a Ca^2+^-independent mechanism involving unidentified RuV receptor(s) [7]. Together with the above findings, this study suggests that it is necessary to continue searching for alternative RuV receptors.

It has been well established that transplacental transmission of rubella occurs in up to 90% of cases during the first eight weeks of gestation, falling to as low as 25% during the second trimester. Though most studies attribute fetal susceptibility to RuV-related teratogenesis in the first trimester of pregnancy to the critical periods of major organogenesis, structural changes in human placental tissues are another factor involved in utero transmission. This fluctuating incidence of fetal infection is likely related to changes in histological and molecular factors associated with placentogenesis. Therefore, considering the finding of this study, an epithelial–mesenchymal transition approach to studying the mechanisms of transplacental vertical RuV infection may be useful.

## 3. Materials and Methods

### 3.1. Cells and Viruses

#### 3.1.1. Cells and Cell Cultures

The human first-trimester trophoblast cell lines Swan71 (derived from telomerase-mediated transformation of a seven-week cytotrophoblast isolate described by Straszewski-Chavez) [8] and HTR8/SVneo (originally obtained from human first-trimester placenta and immortalized by transfection with a cDNA construct that encodes the simian virus 40 large T antigen) [9] were cultured in Dulbecco’s modified Eagle’s medium (DMEM) (Swan71) or Roswell Park Memorial Institute 1640 normal growth medium (RPMI 1640) (HTR8/SVneo) (Gibco-Invitrogen, Tokyo, Japan) supplemented with 10% fetal bovine serum (FBS), 10 mM HEPES, 0.1 mM minimal essential medium non-essential amino acids, and 1 mM sodium pyruvate (Gibco-Invitrogen). HaCaT cells were kindly provided by Dr. N.E. Fusenig (German Cancer Research Center, Heidelberg, Germany) and grown in DMEM containing 10% FBS. Human umbilical vein endothelial cells (HUVECs; Lonza, Tokyo, Japan) were cultured in endothelial growth medium (2% FBS) (EGM-2, Lonza). RK-13 cells were obtained from the Kitasato University School of Medicine (Tokyo, Japan) and were cultured in MEM supplemented with 10% FBS. All the above media were supplemented with 100 IU/mL penicillin and 100 μg/mL streptomycin. The cells were cultured in monolayers at 37 °C in a humidified 5% CO_2_ incubator.

#### 3.1.2. Virus and Virus Infection

RuV strains, both vaccine (Takahashi strain) and clinical (3-B1-RK13 strain), were obtained from Kitasato University School of Medicine (Tokyo, Japan). The viruses were propagated and titered in RK-13 cells.

Cells cultured on glass coverslips in six-well plates (2 × 10^5^ cells/well) were washed with phosphate buffered saline (PBS) and infected with RuV for 1 h at room temperature (RT). After a 1 h adsorption, appropriate medium supplemented with 2% FBS, either RPMI (for HTR8/SVneo cells) or DMEM (for Swan71 and HaCaT cells), was added, and the cells were incubated at 35 °C in a humidified 5% CO_2_ incubator. The next day, the supernatant was removed and replaced with fresh medium (2% FBS). Two days post-infection, the cells were subjected to immunofluorescence assays.

### 3.2. Immunofluorescence Assay

Cells grown on glass cover slips in six-well plates were incubated with RuV as described above. At 48 h post-infection, the supernatant was removed, and the cells were fixed with 4% paraformaldehyde (PFA) solution for 10 min, washed with PBS, and incubated with a mouse monoclonal anti-RuV capsid antibody (ab34749, Abcam, Tokyo, Japan) for 1 h at RT. Negative controls were mock-treated with virus treatment and stained with normal mouse serum. After washing with PBS, the cells were incubated with a FITC-conjugated rabbit anti-mouse IgG (H + L) secondary antibody (Life Technologies, Tokyo, Japan) solution for 30 min at RT. The samples were counterstained with 4′,6-diamidino-2-phenylindole dihydrochloride (DAPI) (Lonza) for nuclear staining. After washing, the cells were mounted with Fluoromount G (SouthernBiotech, Birmingham, AL, USA), and fluorescence images were collected using a fluorescence microscope (Floid Cell Imaging Station; Life Technologies, Tokyo, Japan).

To examine MOG expression, cells grown on glass cover slips in six-well plates for 24 h were fixed with 4% PFA and then subjected to the procedures described above using a rabbit monoclonal anti-MOG antibody (ab109746, Abcam) followed by an Alexa Fluor 488-conjugated secondary antibody.

### 3.3. Western Blotting

Cells grown in six-well plates were incubated with RuV as described above. At 48 h post-infection, the supernatant was removed, and the cells were washed and then lysed in 70 μL of cell lysis buffer (Cell Signaling Technology, Tokyo, Japan). The protein concentrations of the lysates were quantified using DC Protein Assay (Bio-Rad Laboratories, Inc., Hercules, CA, USA). The cell lysates were then loaded onto a NUPAGE 4–12% Bis-Tris protein gel (Invitrogen) and separated by electrophoresis. Following electrophoresis, the proteins were transferred to polyvinylidene fluoride membranes (Invitrogen), and non-specific binding sites were blocked with 1% bovine serum albumin in PBS with 0.1% Tween-20. The membranes were incubated with a mouse monoclonal anti-RuV capsid antibody at 4 °C overnight. The membrane was then incubated with horseradish peroxidase-conjugated secondary antibodies (Abcam) for 30 min at RT and visualized with a Luminescent Image Analyzer, Image Reader LAS-4000 mini (Fujifilm K. K, Tokyo, Japan). To examine MOG expression, cells grown in six-well plates for 24 h were subjected to the procedures described using a rabbit monoclonal anti-MOG antibody (ab109746, Abcam).

### 3.4. Reverse Transcriptase PCR

Total RNA was extracted from cells by using TRIzol (Invitrogen). The RNA concentration was quantified by using a nanodrop spectrophotometer at 260 nm. RT-PCR was performed using PrimeScript One Step RT-PCR Kit Version 2 (Takara Bio Inc., Otsu, Japan) and the following primers: for MOG: sense, 5′-TCC TCC TCC TCC TCC AAG TGT CT-3′; antisense, 5′-AGT GGG GAT CAA AAG TCC GGT GG-3′; for β-actin: sense, 5′-TGG CAC CCA GCA CAA TGA A-3′; antisense, 5′-CTA AGT CAT AGT CCG CCT AGA AGC A-3′. The PCR products were identified by electrophoresis on 1.5% agarose gels.

## 4. Conclusions

This study provides evidence of MOG-independent RuV infection and demonstrates that it is important to continue searching for alternative RuV receptors. As indicated by the susceptibility of HaCaT cells to RuV, this study shows that an immortalized keratinocyte cell line can be used as an in vitro model for future studies of RuV. In addition, this study reports the resistance of first-trimester trophoblast cells to RuV and suggests utilizing an epithelial–mesenchymal transition approach to study the mechanisms of transplacental vertical RuV infection.
